# Control effect of virtual reality technology on procedural pain in children’s wound: A meta-analysis

**DOI:** 10.1097/MD.0000000000030961

**Published:** 2022-10-07

**Authors:** Tuan Li, Yingping Fu, Yanzheng Yang, Yu-E Zhou

**Affiliations:** a Yunnan University of Chinese Medicine, Kunming, Yunnan, People’s Republic of China; b The Third People’s Hospital of Yunnan Province, Kunming, Yunnan, People’s Republic of China.

**Keywords:** children, meta-analysis, nursing, pain, virtual reality technology, wound

## Abstract

Children who undergo wound manipulation usually experience pain. Virtual reality technology is a novel and effective non pharmaceutical therapy for reducing pain in children scheduled to undergo wound manipulation. However, the effectiveness of Virtual reality technology in controlling procedural pain in children’s wounds has not been evaluated in a systematic review.

**Methods:**

It employed a meta-analysis design. We included studies with randomized controlled trials, reporting children’s wound manipulation pain, and published them in English. Two reviewers independently evaluated the methodological quality of the included studies.

**Results:**

Of the 108 studies identified, 39 were eligible for the meta-analysis, with a total sample of 273 patients. The use of virtual reality technology has significantly reduced pain intensity during wound manipulation in children. There was a significant difference between the experimental group (virtual reality) and the control group (no virtual reality) in reducing the pain of the children’s wound manipulation (*P* < .05).

**Conclusion:**

As a distraction method of non drug assisted analgesia intervention, virtual reality technology can reduce children’s procedural pain and discomfort symptoms.

## 1. Introduction

Pain is “a painful experience associated with actual or potential tissue damage including sensory, emotional, cognitive, and social factors”.^[1]^ Pain in children is a subjective, quite unpleasant response of the organism to a variety of external traumatic stimuli. For children, pain has now gradually become the fifth major vital sign after the four major vital signs of body temperature, respiration, pulse, and blood pressure,^[2]^ and is a complex experience that includes sensory, cognitive, behavioral, and psychological factors.^[3]^ Procedural wound pain specifically refers to the changing of dressings, diagnostic or therapeutic interventions, routine nursing care, or physical therapy that cause unpleasant experiences for patients.^[4]^ There is study that show^[5]^: repeated or intense painful stimulation in children leads to a disturbed hormonal secretion, which will create alterations in the structure and function of the organism and has the potential to persist into the adult stage. Current interventions for wound pain in children mainly include distraction therapy, use of anesthetics, cold therapy, etc.^[6]^ Because the children’s immune system is not sound, the ability of tolerance and metabolism is insufficient, so that the skin local anesthetic has a long onset time, which limits the use of anesthetic drugs in children and is not suitable for emergency or emergency situations; The use of cold therapy may cause mild discomfort to children, therefore, currently, more and more studies focus on distraction therapy.^[7]^ Distraction therapy, commonly used for pain management during medical procedures, is an active coping strategy in which patients shift their attention away from noxious stimuli to reduce awareness of pain.^[8,9]^ Distraction tools such as kaleidoscopes, distraction cards, music, and video games, have been shown to be effective in pain management.^[10–12]^ In recent years, many studies have shown^[13–15]^ that the use of virtual reality technology as a distraction therapy can effectively reduce child pain and has been used to manage child pain, including injection procedures, dressings, burns, and chronic and postoperative pain. Virtual reality technology refers to the use of computer technology, constituting a virtual, realistic world, through a certain input or output device, users can participate in the virtual world, form interactions, express their real actions, behaviors, and so on as the control of objects in the virtual world and other behaviors.^[16]^ Hoffman et al^[17,18]^ proposed that the reduction of pain by virtual reality technology is based on a distracting mechanism, the essence of which is that subjects hallucinate themselves to another place, that is, to experience subjectively in a computer-generated world. The experiencer, through a user terminal, such as virtual reality helmet, one-machine, etc, watching the image of the virtual world produced by computer simulation, listening to sounds, feeling movements, etc, performs immersive experiences in the multiple senses of vision, hearing and touch, and interacts with the virtual reality system to achieve their own feelings in reciprocal reactions and feedback,^[19]^ which can help the experiencer actively participate in tasks with cognitive or behavioral functions,^[7]^ Successful distractors stimulate the senses and are highly interactive to draw the attention of the experientia.^[20]^

### 1.1. Background

First applied virtual reality technology to 2 adolescent patients with severe burns at the Washington University Affiliated burn center and showed that the system could not only distract and reduce pain and anxiety, but also promote limb extension of the patients, favoring recovery.^[21]^ Despite active intervention reviews and reports of pain reduction, uncertainties remain about the effectiveness of virtual reality interventions. Some studies^[22–24]^ have shown that although pain is relieved when burn patients use virtual reality assisted physiotherapy, their subjective perception and mobility are not significantly improved.^[25]^ Therefore, the efficacy of virtual reality technology for pain control remains to be further studied. In this study, a meta analytic approach will be used to provide a comprehensive review of the literature on the efficacy of pain control due to children using virtual reality technology for wound manipulation in the hope of providing a more scientific basis for the control of pain in children.

## 2. The review

### 2.1. Aims

We aimed to evaluate the use of virtual reality technology for procedural pain control in pediatric wounds.

### 2.2. Design

We followed the Cochrane criteria Items for Meta-Analysis (PRISMA) guidelines for this meta- analysis.

### 2.3. Search methods

The Chinese knowledge network, Wanfang database, vipu database, Chinese biomedical literature database, PubMed, Embase, Web of Science and Cochrane Library were computationally searched by 2 searchers. Initial keywords included child/children; virtual reality/virtual simulation/virtual environment/Reality, Virtual/Virtual Reality, Educational/Educational Virtual Realities/Educational Virtual Reality/Reality, Educational Virtual/Virtual Realities, Educational/Virtual Reality, Instructional/Instructional Virtual Realities/Instructional Virtual Reality/Realities, Instructional Virtual/Reality, Instructional Virtual/Virtual Realities, Instructional; pain/Ache/Physical Suffering; wound nursing/wound care/wound surface/dressing. The medical subject heading (MeSH) or Emtree terms of each keyword and combinations by using Boolean operators such as “AND” and “OR” were explored in each database. Date range of the search was from database establishment until October 2021. Reference lists of relevant reviews and every included study were hand searched for potential additional studies.

### 2.4. Search outcomes

**Figure 1.**
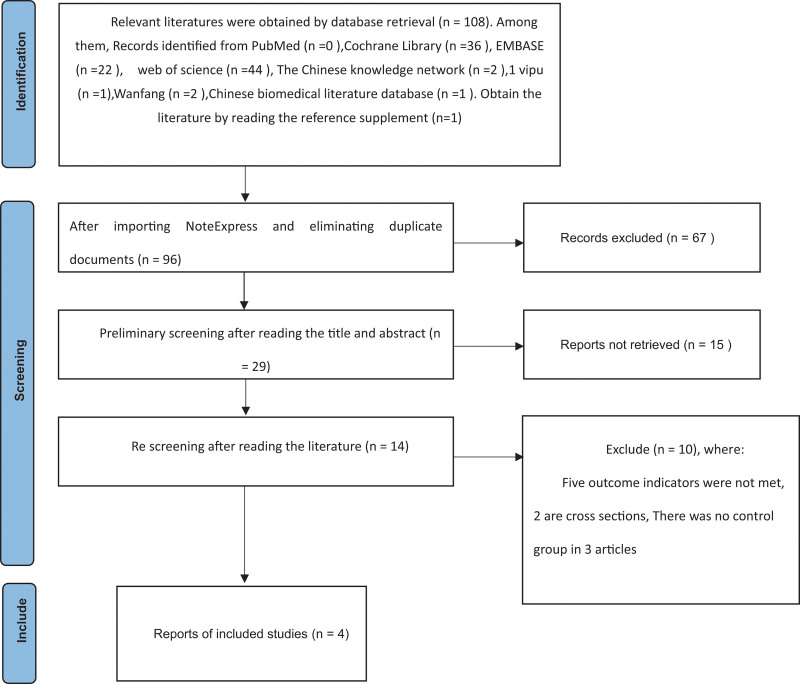
The box indicates the processing to be executed, and the arrow indicates the direction of program execution. This diagram uses the template from PRISMA 2020 flow diagram and appears in the style specified by the original copyright holder.

The literature search was performed independently by 2 researchers, and those that did not meet the inclusion criteria were excluded by initial screening by reading the literature titles and abstracts, and then by reading the full text check. In case of disagreement, inclusion was decided by arbitration by the third researcher or by discussion with the study team. The Preferred Reporting Items for Meta-analysis flow diagram^[26]^ was used to illustrate the study selection (refer to Fig. 1). The inclusion criteria were: study type: randomized controlled trial. Study subjects: children (same as *The Convention on the rights of the child*, which refers to anyone under the age of 18 years) who practice manipulation of wounds voluntarily participated in this investigation. Interventions: 2 groups were divided between experimental and control groups, the experimental group received only virtual reality technology, and the control group did not have virtual reality technology intervention for analgesia. Literature interventions with clear results, outcome measures have Wong-Baker facial expression scale, visual analogue scale (VAS), face, legs, activity, crying, consolability (FLACC) pain behavior scale, pulse rate, blood oxygen, saturation long-lasting, adverse effects. The language of publication was Chinese or English. The exclusion criteria were: duplicate published literatures, literatures without valid data or whose full texts could not be obtained by various methods. Studies combining other interventions. The literature was of too low quality, lacked outcome measures, or had significant study flaws.

### 2.5. Quality appraisal

According to the Cochrane Handbook for systematic reviews 5.1.0 criteria for reviews,^[27]^ the included literature was independently assessed for quality by 2 researchers. Evaluation items included: generation of random sequence, allocation concealment, double blinding of implementers, and participants, blinding of outcome assessors, completeness of outcome data, selective reporting of outcomes, and other sources of bias. The quality of the literature was classified into 3 levels, A, B, and C. Grade A: fully meeting the above criteria with likely minimal occurrence of bias; Grade B: partially meeting the above criteria, occurrence of bias may be moderate; Grade C: does not meet the above criteria at all and occurrence of bias may be high. Disagreements were adjudicated by a third investigator or resolved by discussion of the study team.

### 2.6. Data abstraction

Data from included studies were independently extracted by three researchers. The extracted data included: methodological information on the studies: first author, year of publication, region, included subjects, age, number of study cases, type of virtual reality; reported outcome measures. Wong-Baker facial expression scale. The method employs 6 facial expressions describing pain in different expressions ranging from smiling to crying. The meaning of each expression representation was first explained to the children. 0: very pleasant, no pain; 2: A little pain; 4: Minor pain; 6: Pain was more pronounced; 8: More severe pain; 10: Severe pain. The lighter the pain in a closer left expression, the more severe the pain in a closer right expression. Children were then asked to indicate which expression best represented the level of pain.^[28]^ VAS. This scale, used in children over 6 years of age, often employs a 10 cm long straight line, with both ends denoting “no pain” (0) and “worst pain imaginable” (10), respectively. Children can mark the intensity of their pain and how well they feel psychologically, at some point on the straight line, based on the level of pain they experience. The length of distance from the origin to the marker is also the amount of pain.^[29]^ FLACC pain behavior scale. Mainly suitable for age 0 to 3 years. Five items were included, and each item was rated from 0 to 2, with a minimum score of 0 and a maximum score of 10 on the total scale, with higher scores indicating greater discomfort and pain.^[30]^

### 2.7. Synthesis

Meta-analysis of data from included studies was performed using Revman 5.4 statistical software. A weighted mean difference analysis using the same measurement tools as in the text; different measurement tools were then analyzed using the standardized mean difference. There were analyses in which the 95% confidence interval (CI) was calculated to determine whether there was heterogeneity among studies by *I*^2^, if *P* values >.1, *I*^2^ < 50% indicated no heterogeneity among studies, the fixed effect model was selected for analysis; If *P* values ≤.1, *I*^2^ ≥ 50% indicated that there was heterogeneity among studies, random effects model was selected for analysis.

## 3. Results

### 3.1. Identification and selection of studies

A total of 108 articles were obtained from the initial search, including 102 in English, 6 in Chinese, and 1 additional included articles when reading the references of relevant articles. There were 96 publications after importing NoteExpress to remove duplicates. After reading the titles and abstracts, 29 remaining articles that did not meet the inclusion criteria were excluded. After searching the full texts for reading, 15 literatures were further excluded. Ten were excluded by careful reading again, of which 5 outcome measures were not met; 2 articles were cross-sectional studies; 3 had no control group. Four literatures were finally included.^[31–34]^ A total of 273 patients were included, 136 in the experimental group and 137 in the control group.

### 3.2. Characteristics of the studies

General conditions of included studies are shown in Table 1. The included trials were prospectively registered. A total of 4 randomized controlled trials were included in this study, all of which described the method of randomization, 3 papers used computer-generated random numbers, and 1 literature generated random sequences according to the order of visits; 2 literature articles reported the allocation concealment scheme, and the remaining 2 did not mention related content. Due to the inability of virtual reality to implement blinding, subjects and intervention implementers blinding in 4 literatures were evaluated as high risk of bias. No literature specifically described the use of an intention to treat analysis approach to guarantee the completeness of outcome measures; The 2 articles specifically described the reasons for participants’ withdrawal from the study. Blinding of outcome assessors was explicitly proposed in 2 articles, whereas none of the 2 literature authors mentioned it. Objective physiological parameters such as pulse rate and oxygen saturation were included in the 2 studies. The presence of other factors causing bias could not be assessed based on the information provided by the included studies, so the other biases were all unclear (omitted in the results). Of the 4 articles, 3 had a grade B and 1 had a grade C quality. Specific evaluation indicators and outcomes are shown in Table 2 (assessment category as risk of bias).

**Table 1:** Basic characteristics of the included study.

Inclusion study	Year of publication	Region	Included object	Age	Number of cases		Intervention measures		Types of virtual reality technology	Outcome indicators
					experience group	control group	experience group	control group		
Wu Yujie et al	2020	China	Dressing change of infected wound	5–18 years old	48	48	A	B	Virtual reality glasses suitable for playing virtual video	①④⑥⑦
Remziye et al	2020	Turkey	Venous access in tumor patients	7–18 years old	35	36	A	B	Virtual reality helmet	①
Belinda et al	2012	Australia	Burn dressing change	11–18 years old	20	21	A	B	Virtual reality system, age appropriate software game	②③④⑥⑦
Yun Hua et al	2015	China	Dressing change of chronic wound of lower extremity	4–16 years old	33	32	A	B	Ice age 2 video games, computers and head mounted displays	①②③④⑤⑥

Note: A is the virtual reality technology intervention, B is the control (no virtual reality technology intervention) ① Wong Baker facial expression scale ② visual analogue scale ③ FLACC pain behavior scale ④ pulse rate ⑤ blood oxygen saturation ⑥ dressing change duration ⑦ adverse reactions

**Table 2:** Risk of bias for included studies

Inclusion study	Year of publication	Random sequence generation	Assign hide	Double blind for implementers and participants	Outcome evaluator blind method	Data integrity of outcome indicators	Publication bias	Document quality grade
Wu Yujie et al	2020	High	unclear	High	High	low	low	C
Remziye et al	2020	low	low	High	High	low	low	B
Belind et al	2012	low	low	High	unclear	low	low	B
Yun Hua et al	2015	low	unclear	High	unclear	low	low	B

### 3.3. Meta-analysis

#### 3.3.1. Effect on pain scores

##### 3.3.1.1. Wong-baker facial expression scale

**Figure 2.**
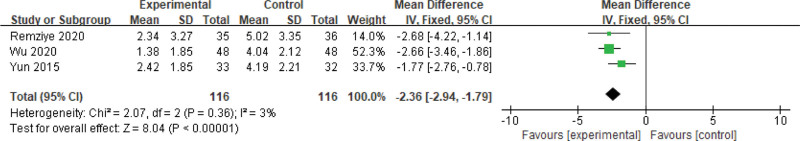
Mean refers to arithmetical mean, and SD refers to standarddeviation. CI refers to confidence interval, and 95%CI means that 95% ci is equivalent to the hypothesis test of а = 0.05. Weight refers to the weight of each effect value, the length of horizontal line represents the confidence interval range, the size of square represents the weight (the contribution of this research to meta-analysis), the diamond represents the combined effect quantity, and the vertical solid line in the figure represents the invalid line, which is used to judge whether the difference of results is statistically significant.

Three studies reported the use of virtual reality on the scoring of the Wong Baker facial expression scale and there was no heterogeneity among studies (*P* = .36, *I*^2^ = 3%) a fixed effect model was used for analysis. The results showed that the Wong Baker facial expression scale score of the test group was lower than that of the control group after the intervention using virtual reality, with a statistically significant difference (MD = –2.36, 95% CI: –2.94 to –1.79, *P* < .001, Fig. 2).

##### 3.3.1.2. Visual analogue scale

**Figure 3.**
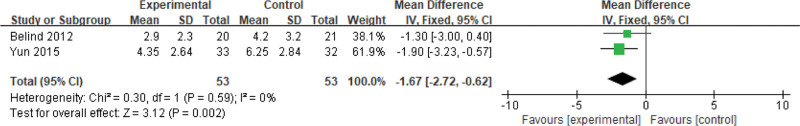
Each part/panel has the same meaning as that of Figure 2.

Two studies reported the effect of using virtual reality on visual analogue scale pain conditions, and there was heterogeneity across studies (*P* = .59, *I*^2^ = 0%) using a fixed effects model for analysis. The results showed that the VAS score of the experimental group was lower than that of the control group after the adoption of virtual reality, and the difference was statistically significant (MD = –1.67, 95% CI: –2.72 to –0.62, *P* < .001, Fig. 3).

##### 3.3.1.3. FLACC pain behavior scale

**Figure 4.**
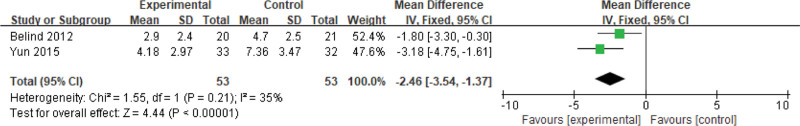
Each part/panel has the same meaning as that of Figure 2.

Two studies reported the effect of virtual reality technology on the score of FLACC pain behavior scale. There was heterogeneity among the studies (*P* = .21, *I*^2^ = 35%), which was analyzed by selective fixed effect model. The results showed that after the intervention with virtual reality technology, the score of FLACC pain behavior scale in the experimental group was lower than that in the control group, and the difference was statistically significant (MD = –2.46,95% CI: –3.54 to –1.37, *P* < .001 Fig. 4).

#### 3.3.2. Pulse rate

**Figure 5.**
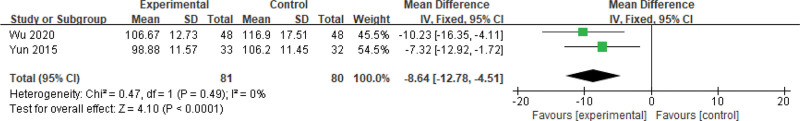
Each part/panel has the same meaning as that of Figure 2.

Two studies reported the impact of virtual reality technology on pulse rate. There was heterogeneity among studies (*P* = .49, *I*^2^ = 0%), which was analyzed by selective fixed effect model. The results showed that after using virtual reality, the pulse rate of the experimental group was lower than that of the control group, and the difference was statistically significant (MD = –8.64,95% CI: –12.78 to –4.51, *P* < .001, Fig. 5).

## 4. Discussion

### 4.1. Effect of virtual reality therapy on wound procedural pain

The heterogeneity of the literature included in this meta-analysis is low. The results show that virtual reality technology, as a non drug assisted analgesic intervention, can reduce the operative pain of patients’ wounds, reduce the pulse rate, reduce the length of dressing change and adverse reactions. A study^[35]^ compared the effects of simple analgesia and analgesia plus virtual reality technology on children during burn wound care. The results showed that analgesia combined with virtual reality technology can reduce pain more effectively than simple analgesia. Studies^[36]^ have shown that virtual reality technology can effectively alleviate the pain caused by tooth extraction by distracting patients’ attention. After applying this therapy to 60 children, the research results are consistent.^[37]^ Chen et al^[38]^ showed that virtual reality technology for 36 children aged 7 to 12 who underwent venipuncture in the emergency room can effectively reduce the degree of pain. Le may et al^[39]^ found that virtual reality technology can shorten the treatment time and improve work efficiency.

### 4.2. Implications of this meta-analysis for future research

The application of virtual reality technology is still in its infancy, and there are still many deficiencies: the sample size involved in foreign clinical trials and laboratory research is small, and the sample population is limited. It is generally divided into burn patients,^[40]^ cancer patients, etc, and there are few studies used to control the pain of other types of people; There are few intervention studies on pain relief using virtual reality technology in China, most of which are theoretical reviews.^[6,41,42]^ When evaluating pain, patients’ subjective scores are often used as the basis, such as Wong Baker facial expression scale and visual analog scale, which lack the evaluation of objective indicators. In the future, biofeedback system and emotion recognition system can be used to accurately identify the physiological and emotional changes of human body and objectively judge the effectiveness of experimental results. Because children of different ages will show different cognitive characteristics and behaviors, and will show different coping styles to virtual reality technology, it is necessary to fully consider the individual differences of children. The subjects included in this meta-analysis are 4 to 18 years old, and most studies have less relevant intervention for children under 4 years old. It is suggested that future studies can evaluate and intervene children of different ages. In addition, virtual reality technology has the limitations of high cost, software development, and hardware accessibility. Therefore, when designing virtual reality technology system, it is still an area to be further studied to meet the needs of patients, reflect the color of personalization and humanistic care, and meet the requirements for the hardware level of virtual equipment and provide the most suitable application program for the treatment effect. Inserting parents’ comforting words or parents’ smiling faces in the virtual reality picture can give children emotional support to reduce insecurity, and also reduce the obstruction of parents’ company to the medical staff in the treatment process.^[43]^ Virtual reality is difficult to achieve blind intervention, and further high-quality research is still needed to verify the research results in the future.

### 4.3. Limitations

Only published Chinese and English literatures were retrieved in this study, which did not contain relevant studies in other languages, and publication bias may exist. As the interventions could not be blinded and the nurses performing the outcome measures were likely to have provided more careful care to the patients in the trial groups when the grouping was known in advance, the double-blind design, although challenging, is an ideal design for future studies.

## 5. Conclusion

This meta-analysis analyzed the effect of virtual reality technology as an auxiliary analgesic distraction therapy on wound procedural pain from the perspective of evidence-based. The results show that virtual reality technology can effectively reduce children’s wound procedural pain and discomfort symptoms, but the sample size is small and the sample population is limited. Nevertheless, we can still think that virtual reality technology is an effective distraction therapy, and look forward to carrying out more scientific and rigorous design in the future to provide a stronger evidence-based basis for verifying its potential advantages.

Mastera, Mastera, Mastera,

## Author contributions

**Conceptualization:** Tuan Li.

**Data curation:** Tuan Li, Yingping Fu.

**Formal analysis:** Tuan Li, Yu-E Zhou.

**Methodology:** Yu-E Zhou.

**Software:** Yanzheng Yang.

**Validation:** Yanzheng Yang.

**Writing – original draft:** Tuan Li, Yingping Fu.

**Writing – review & editing:** Tuan Li, Yu-E Zhou.
